# Shifting the centre of gravity in the global evidence ecosystem for health: strengthening local leadership and decision-making for national and global impact

**DOI:** 10.1136/bmjgh-2025-020093

**Published:** 2025-12-25

**Authors:** Tanja Kuchenmüller, Sandy Oliver, Laurenz Mahlanza-Langer, Holger Schünemann, Annette Boaz, Peter Kasadha, Fadi El-Jardali, Mukdarut Bangpan, Donald Simeon, Shelly-Ann Hunte, Yodi Mahendradhata, Firmaye Bogale, Veronica Osorio Calderon, Mandip Aujla, Mareike Günther, Marge Reinap, Ludovic Reveiz, Arash Rashidian, Mehrnaz Kheirandish, Tarang Sharma, John C Reeder, Jeremy Farrar, Laura dos Santos Boeira

**Affiliations:** 1 Science Division, WHO, Geneva, Switzerland; 2 EPPI-Centre, Social Science Research Unit, UCL Social Research Institute, University College London, London, England, UK; 3 Pan-African Collective for Evidence, Johannesburg, South Africa; 4 Humanitas University, Milan, Italy; 5 NIHR Health and Social Care Workforce Research Unit, King’s College London, London, UK; 6 Center for Rapid Evidence Synthesis, Kampala, Uganda; 7 Knowledge to Policy Center/WHO Collaborating Centre for Evidence-Informed Policymaking and Practice, and Department of Health Management and Policy, American University of Beirut, Beirut, Lebanon; 8 Caribbean Centre for Health Systems Research and Development, The University of the West Indies, St. Augustine, Trinidad and Tobago; 9 Faculty of Medicine, Public Health and Nursing, Universitas Gadjah Mada, Daerah Istimewa Yogyakarta, Indonesia; 10 Ethiopian Public Health Institute, Addis Ababa, Ethiopia; 11 Division of Country Health Policies and System, WHO Regional Office for Europe, Copenhagen, Denmark; 12 Science and Knowledge for Impact Unit, Department for Evidence and Intelligence for Action in Health, Pan American Health Organization, Washington, District of Columbia, USA; 13 Department of Science, Information and Dissemination, WHO Regional Office for the Eastern Mediterranean, Cairo, Egypt; 14 Instituto Veredas, São Paulo, SP, Brazil

**Keywords:** Global Health, Health policy, Interdisciplinary Research

**SUMMARY BOX**

- Global challenges in recent years, including humanitarian, climate-related and public health crises, have highlighted critical weaknesses in the application of evidence in decision-making, both locally and globally.
- The Global Coalition for Evidence connects efforts made around the world and empowers local leadership to address the fragmentation and inequities in the health evidence ecosystem.
- By adopting the ‘3Cs’ approach, the Global Coalition for Evidence promotes collaboration, coordination and consolidation to streamline efforts and institutionalise evidence use for better health outcomes.

While the importance of evidence for decision-making has been well-documented, global challenges in recent years, including the COVID-19 pandemic, humanitarian crises, climate change and the associated health disparities and inequities, have exposed critical weaknesses in the global evidence architecture.^1 2^ The announcement of funding for global living evidence syntheses by major donors at the Summit of the Future in September 2024,^3^ followed by the Cape Town Consensus Statement and renewed commitments in the context of the United Nations General Assembly 2025^4^, collectively signal a shared commitment to elevating the role of evidence in societal progress. The launch of the Global Coalition for Evidence at the Global Evidence Summit 2024 further advanced this agenda by fostering collaboration to promote systematic and transparent evidence use at the country level.

In this commentary, the founding group of the coalition explores key challenges and opportunities to strengthen evidence-informed decision-making (EIDM) in health. We argue that efforts to unify the global evidence ecosystem to support countries must prioritise principles of equity and solidarity, especially in the current geopolitical context, with so many potential issues transcending borders.^5 6^

National policymaking entails navigating the politics of decision-making, balancing competing political and societal priorities, while addressing resource constraints and inevitable complex trade-offs. Major barriers to EIDM include a lack of local capacities to translate and systematically use different forms of evidence regularly and in real time; a lack of adherence to systematic and transparent processes and standards and limited infrastructures to support the uptake and application of evidence.^7^ The gap between evidence and policy can be further exacerbated by misaligned national policy and research production cycles and compounded by insufficient investments in national research systems, which are vital for producing policy-relevant research.

To strengthen and embed mechanisms that contribute to greater routine use of evidence in decision-making, a deliberate shift in power dynamics is needed, which enables national leaders to take ownership of the EIDM process in their institutions. This process should promote consideration and translation of global evidence syntheses to the national context and their use to address local policy priorities. To achieve this, stronger institutional capacity, enhanced decision-making processes, targeted funding and inclusive governance structures that support locally driven solutions are necessary.^8^ In response to this need, the Global Coalition for Evidence, convened and supported by the WHO, aims to foster equitable and reciprocal partnerships, strengthen capacities and ensure the sustainable integration of evidence into policymaking frameworks to improve health outcomes at national, regional and global levels.

Acknowledging the interconnectedness and interdependence of national and global evidence ecosystems, as well as entrenched power dynamics within them, the Coalition is adopting a ‘3Cs’ approach based on collaboration, coordination and consolidation to create more effective, sustainable solutions to local health and social priorities. Anchored in SDG3 and a Health in All Policies perspective, this approach spans all levels of governance—from local to global—and aims to reduce fragmentation and foster inclusive processes by engaging a broad range of interest-holders, including policymakers, implementers, advocates, researchers and civil society.

## Collaboration

It is crucial to enhance collaboration to address complex, intranational and transnational health challenges, such as climate change or Planetary Health, and tackle ongoing struggles for health services in low-resource settings. The coalition will cultivate a collaborative culture built on equity and trust and promote networking, knowledge exchange and joint learning for social change. It will draw on the experience of global initiatives such as WHO’s Evidence-informed Policy Network and regional efforts such as the Pan-African Collective for Evidence, the Latin American and the Caribbean Evidence Hub and the Knowledge to Policy Centre, with the mandate to strengthen local institutional EIDM capacities.

## Coordination

A disjointed evidence ecosystem with siloed workstreams and sectors hinders effective EIDM and increases the risk of duplication of efforts and waste of limited resources.^9^ The coalition seeks to improve the coordination and integration of evidence workstreams such as evidence syntheses, guideline development or adaptation, health technology assessment (HTA) reports and data analytics to address systemic bottlenecks and create opportunities to pool resources. To ensure complementarity, the coalition also aims to coordinate work across countries and agencies, for example, between Cochrane and its production of high-quality evidence syntheses and methodological innovations and the Guideline International Network or Health Technology Assessment International.

## Consolidation

The proliferation of independently developed EIDM tools, evidence products and guidelines risks resulting in overlapping, contradictory approaches that confuse users and potentially lead to normative inconsistencies.^9^ The coalition will work to consolidate global efforts and integrate them into cohesive frameworks that guide choices. Two examples that the coalition will build on are the GRADE (Grades of Recommendation, Assessment, Development and Evaluation) working groups and their role in developing evidence-to-decision frameworks, and the Global Research Agenda on knowledge translation and evidence-informed policy, consolidating priority research areas on research use.

The coalition recognises that special attention is needed for low- and middle-income countries (LMICs) and fragile settings, where timely, context-relevant decisions depend on accessible and reliable evidence. In many such settings, core EIDM functions—such as HTA—are underdeveloped, poorly structured and underfunded. It seeks to address these gaps by improving access to evidence and promoting systematic approaches to judicious evidence use that are sensitive to differences in context.

The coalition employs four evolving thematic working groups, shown in table 1, which form the foundation for its future direction. In recent months, the working groups initiated key conceptual work, including the launch of the Global Research Agenda on Knowledge Translation and Evidence-Informed Policy-Making to guide future research in this field.

**Table 1:** Working groups of the global coalition for evidence

Working group 1: EIDM institutionalisation and strengthening national evidence-for-policy systems	Working group 2: integrating different evidence workstreams
Deepen knowledge and integrate learning from tested tools for supporting EIDM institutionalisation, mobilise policymakers to document and share their evidence-use practices and collate measures and tools to assess evidence-use among policymakers.	Understand barriers and enablers for strengthening the health decision-making ecosystem, develop a comprehensive framework for better coordination across the evidence ecosystem and draft a blueprint and roadmap to pilot and operationalise this framework.

Working group 3: setting priorities and promoting research on research use	Working group 4: promoting collaborative learning for linking evidence and decisions
Identify global KT and EIDM research priorities to improve efficiency and synergy for research on research use, enhance collaboration across health sectors and raise awareness about KT/EIDM research and improve understanding of approaches in the use and synthesis of EIDM.	Understand existing knowledge about collaborative learning and social change, reflect on how these processes occur within and beyond the coalition and document the consequences of working collaboratively for learning and change.

Source: 10

EIDM, evidence-informed decision-making; KT, knowledge translation.

There is currently strong momentum to bring together the different actors involved in producing, translating and using health evidence through equitable collaboration, coordination and consolidation to improve health outcomes. Designed as a long-term platform rather than a temporary, project-based initiative, the coalition will span intersectoral boundaries, facilitate the joining of fragmented knowledge and resources, enhance shared commitment and integrate tacit insights and lessons learnt.

The next step will involve translating the coalition’s momentum into concrete action. To this end, key priorities include^1^ establishing inclusive governance approaches and promoting coordinated research to ensure partner ownership, accountability and improved understanding of evidence use^2^; securing sustained, flexible funding to advance the coalition’s work^3^; embedding evidence use in policy cycles through strengthened institutional mechanisms, particularly in LMICs and fragile settings and^4^ expanding the coalition and fostering synergies with existing initiatives through transparent, inclusive processes. Continued efforts to strengthen global-local links and align with SDG3 and broader development goals will be essential to the coalition’s success.

## Data Availability

There are no data in this work.
